# Medical Students and SARS-CoV-2 Vaccination: Attitude and Behaviors

**DOI:** 10.3390/vaccines9020128

**Published:** 2021-02-05

**Authors:** Bartosz Szmyd, Adrian Bartoszek, Filip Franciszek Karuga, Katarzyna Staniecka, Maciej Błaszczyk, Maciej Radek

**Affiliations:** 1 Department of Neurosurgery, Spine and Peripheral Nerves Surgery of Medical University of Lodz, 90-549 Łódź, Poland; bartoszmyd@gmail.com (B.S.); filip.karuga@stud.umed.lodz.pl (F.F.K.); katarzyna.staniecka@stud.umed.lodz.pl (K.S.); maciej.blaszczyk@umed.lodz.pl (M.B.); maciej.radek@umed.lodz.pl (M.R.); 2 Department of Pathophysiology, Medical University of Lublin, 20-090 Lublin, Poland; 3 Department of Sleep Medicine and Metabolic Disorders, Medical University of Lodz, 92-215 Łódz, Poland

**Keywords:** COVID-19, SARS-CoV-2, vaccination, healthcare workers, depression, anxiety, stress

## Abstract

Since physicians play a key role in vaccination, the initial training of medical students (MS) should aim to help shape their attitude in this regard. The beginning of vaccination programs against severe acute respiratory syndrome coronavirus 2 (SARS-CoV-2) is an excellent time to assess the attitudes held by both medical and non-medical students regarding vaccination. A 51- to 53-item questionnaire including the Depression, Anxiety and Stress Scale was administered to 1971 students (49.21% male; 34.86% MS); two career-related questions were also addressed to the MS. The majority of surveyed students indicated a desire to get vaccinated against SARS-CoV-2, with more medical than non-medical students planning to get vaccinated (91.99% vs. 59.42%). The most common concern about SARS-CoV-2 infection was the risk of passing on the disease to elderly relatives. While conspiracy theories regarding the COVID-19 vaccine are less popular among MS, both groups indicated concerns that vaccines may cause autism is equally common (~5%). Further studies exploring social attitudes towards the SARS-CoV-2 vaccine are a necessary first step to optimizing vaccination programs and achieving herd immunity.

## 1. Introduction

As physicians play a key role in vaccination, the initial training of medical students (MS) should not only provide accurate medical knowledge but should also help shape attitudes regarding the topic [1]. Unfortunately, nearly a third of final-year MS feel inadequately prepared to deal with the clinical aspects of vaccination, especially communication issues with vaccine-hesitant patients [1]. This may well be a product of the excessive focus on theoretical knowledge typified in educational programs and the resulting lack of practical skills [2]. This limitation of current medical training is highlighted by the fact that the Internet is considered to be a better source of information about vaccines than medical studies (59% vs. 52% of MS, respectively) [3].

Other significant determinants of vaccine uptake include the clinical years of medical study and any previous history of recommended vaccination (e.g., against influenza) [1,4,5]. The current public debate about vaccination against severe acute respiratory syndrome coronavirus 2 (SARS-CoV-2) is not only a good opportunity to assess student attitudes and behaviors toward vaccination, but also to examine the psychological environment of the students resulting from this situation, such as any depression, anxiety, and stress symptoms. Therefore, the present study attempts to identify any relationship between the aforementioned factors, as well as the current state of knowledge regarding vaccination among students, and their readiness to become vaccinated against SARS-CoV-2. We hypothesize that:

- medical studies form pro-vaccination behaviors;
- depression, anxiety, and stress related to the public debate on vaccination increase the willingness to get vaccinated.

## 2. Materials and Methods

### 2.1. Study Design and Participants

An online survey was performed among Polish medical (MS) and nonmedical students (NMS; control group). NMS comprised all students except those of dentistry, dietetics, emergency medical services, laboratory diagnostics, medicine, nursing, obstetrics, pharmacy, and physiotherapy students. The self-administered online questionnaire was available on Google Forms between 22 and 25 December 2020, during the public debate on SARC-CoV-2 vaccination, before the first vaccination rollout in Poland on 27 December 2020 (including healthcare workers and students of medical faculties). All MS were invited to take the survey through institutional emails, university-affiliated websites, and social media profiles (Facebook). However, as the authors only had access to the institutional emails of students from the Medical University of Lodz (MS), NMS were invited only through social media.

### 2.2. Measurement Tools

A three-part questionnaire was prepared. The first part concerned general information such as age, gender, income, professional status, the population size of the place of residence and work, previous SARS-CoV-2 infection in participants themselves or their relatives, fear of getting infected, willingness to get vaccinated against SARS-CoV-2, and past medical history of mandatory and recommended vaccinations. The second part focused on their grades in microbiology, clinical immunology, introduction to pediatrics, and infectious diseases courses. The final part included the standardized Depression, Anxiety and Stress Scale-21 Items questionnaire (DASS-21) to measure the intensity of depression, anxiety, and stress. As the DASS-21 scale encompasses the previous week, and the MS were due to receive vaccination from 27 December 2020, the study was performed one week before this date. Each item was rated on a 0–3 scale, so that the total scores ranged from 0 to 63. A higher score suggests more severe depression, anxiety, and/or stress symptoms. The scale is widely used in the Polish population [6]. The internal consistency of the DASS-21, assessed with Cronbach’s α, was sufficient for the study (0.921).

### 2.3. Data Collection

All participants were aware of the study conditions and gave informed consent to participate. Confidentiality and anonymity were maintained and no data that could help identify a responder were collected. The local Bioethics Committee confirmed that, according to Polish law and Good Clinical Practice regulations, the study does not require the approval of a Bioethics Committee (KB nr 542/20) [7].

### 2.4. Sample Size Calculation and Statistical Analysis

The sample size calculation was performed using a calculator provided by The University of British Columbia [8]. The frequency of students who wish to be vaccinated was calculated separately for MS and NMS (94% and 70%, respectively) based on the first fifty questionnaires. To provide a standard α = 0.05 and β = 0.80, the minimum size of each group was 40.

The collected data were verified for completeness, quality, and consistency as described previously [7]. Statistical analysis was performed using STATISTICA 13.1 (TIBCO, Palo Alto, Santa Clara, CA, USA). A level of 5% was used as a significance threshold for all results unless stated otherwise. The distribution of the obtained data was assessed by the Shapiro-Wilk test (*p* > 0.05 for normal distribution). Data with a normal distribution are reported as mean with standard deviation (SD), while non-normal data are presented as median with IQR (1. quartile–3. quartile) [9,10]. The relationship between two independent subgroups was assessed using Student’s t-test for normally distributed data, or the Mann-Whitney U-test for non-normally distributed data. The qualitative data were analyzed using the chi-square test, Yates-corrected chi-square or Fisher’s exact test based on the size of the smallest subgroup (*n* ≥ 15, 15 > *n* ≥ 5, 5 > *n*, respectively) [11]. All qualitative findings are presented as *n* (%). Finally, binary logistic regression was performed to properly evaluate the eventual facilitators/barriers affecting the willingness to be vaccinated as soon as possible.

## 3. Results

### 3.1. Study Group

Our study group consisted of 1971 students: 687 (35.23%) MS and 1284 (56.70%) NMS. Their median age was 21 (20–24) and 20 (19–22), respectively. Most of them grew up in the countryside and/or small cities (<50,000 residents) and studied in cities of over 500,000 residents. Details are presented in Table 1.

### 3.2. Experiences with COVID-19 and Related Anxiety

Although MS were tested for SARS-CoV-2 infection significantly more often than NMS, i.e., 296 (43.1%) vs. 197 (15.34%) (*p* < 0.001), the frequency of positive results was similar in both groups: 59 (8.59%) vs. 95 (7.39%) (*p* = 0.349). The frequency of family members with confirmed SARS-CoV-2 infection and those deceased in the course of COVID-19 were similar in both groups (~58% and ~6.9%, respectively). Concerns about infecting elderly relatives were more common in MS than NMS, but only among those with a negative past medical history of SARS-CoV-2 infection. As a result, MS did not visit their elderly relatives or were significantly more likely to restrict their visits to at most once per month, compared to NMS: 398 (58.36%) vs. 656 (51.7%) (*p* = 0.004). The main COVID-19-related concerns of both MS and NMS include health deterioration in family members, post-COVID syndrome, and their own health deterioration. More detailed information about experiences with COVID-19 and related anxiety among MS and NMS are given in Table 2.

### 3.3. The Vaccination-Related Experiences

MS are less worried about vaccination side-effects than NMS: 2 (1–5) vs. 4 (1–7) on a 10-point scale (*p* < 0.001). Most of the students participating in the study declared a desire to get vaccinated against the SARS-CoV-2 virus with messenger ribonucleic acid vaccines [12]. Significantly more MS than NMS would like to get vaccinated rather than not (*p* < 0.001), and as soon as possible rather than at some point in the future (*p* < 0.001). Among both MS and NMS students, the greatest concern regarding long-term complications, followed by fever and malaise for MS and conspiracy theories for NMS (especially the belief that vaccination programs limit civil rights and the possibility of microchip injection). This vaccination seems to be more widely accepted than other recommended vaccinations, such as the influenza vaccine, among both MS and NMS (*p* < 0.001). The vaccination-related experiences among MS and NMS results are collected in Table 3.

### 3.4. The DASS-21 Questionnaire Results

The DASS-21 questionnaire revealed that as compared to NMS, the MS group demonstrated a lower intensity of depression symptoms (6 (3–9) vs. 6 (3–10), *p* = 0.009) but a higher intensity of both anxiety (3 (2–6) vs. 3 (1–6), *p* = 0.035) and stress symptoms (7 (4–10) vs. 6 (4–9), *p* < 0.001) during the public debate about SARS-CoV-2 vaccination (Table 4A). Binary logistic regression indicated that the willingness to get vaccinated as soon as possible was significantly amplified by stress level and reduced by depression symptoms in the previous week.

### 3.5. Factors Influencing Pro-Vaccination Attitudes

In both groups, the desire for early vaccination against SARS-CoV-2 was influenced by the following factors: fear of contracting COVID-19 (*p* < 0.001), concerns about passing it on to close ones (*p* < 0.001), concerns regarding adverse reactions (*p* < 0.001), severity of related anxiety (*p* = 0.002), and stress severity in the preceding week (*p* < 0.001). A positive past medical history of recommended vaccinations (e.g., for influenza) appears to increase the willingness to get vaccinated (*p* < 0.001) and as soon as possible (*p* < 0.001). Neither the severity of depression symptoms in the preceding week (*p* = 0.957) nor monthly expenses (*p* = 0.137) influenced the desire for early vaccination.

Among MS, the year of study affects the desire to get vaccinated: the median of the study year of students who wanted to get vaccinated as soon as possible was year three (2–5) compared to year two (1–4; *p* < 0.001) in the MS preferring to get vaccinated in a different year. However, in the MS group, grades obtained in courses where vaccinology played a key role did not significantly differentiate between those wanting to get vaccinated as soon as possible and those who did not, these courses being microbiology (*p* = 0.877), infectious diseases (*p* = 0.743), immunology (*p* = 0.857), and introduction to pediatrics (*p* = 0.524).

Binary logistic regression indicated that the willingness to get vaccinated as soon as possible is significantly amplified by fear of passing on the disease to relatives (OR = 1.255, 95%CI: 1.113–1.413, *p* < 0.001) and the year of medical study (OR = 1.270, 95%CI: 1.110–1.451, *p* < 0.001). However, fear of vaccination side effects (OR = 0.616, 95%CI: 0.562–0.675, *p* < 0.001) and depression symptoms (OR = 0.930, 95%CI: 0.867–0.997, *p* = 0.043) in the previous week reduce vaccination readiness. Other parameters used in this analysis are collected in Table 5.

## 4. Discussion

This is one of the first studies to assess student attitudes regarding SARS-CoV-2 vaccination during the public debate concerning the safety and necessity of vaccination [13,14].

### 4.1. Medical Education Regarding Vaccinology

Healthcare workers are responsible for providing necessary information regarding vaccine safety and dispelling doubts concerning its use. Therefore, an appropriate level of knowledge in the field of vaccinology is of great importance [1,15,16,17]. Previous medical literature indicates that despite vaccinology being part of mandatory academic subjects in the medical school curriculum, most students do not consider their knowledge sufficient [1,17]. This is supported by a study on MS in France [1]. The lack of knowledge regarding vaccination hence appears to be a large-scale problem affecting higher education across the whole of Europe [1,17].

A study of MS by Kernéis et al. found that 66% of the respondents considered their knowledge of immunology and infectious diseases insufficient [1]. They also demonstrated poor practical skills such as screening for contraindications, knowledge of routes and sites of administration, and management of adverse reactions [1], and were uncertain about the practical aspects of vaccine costs and reimbursement, adjuvant mechanism of action and potential adverse effects, and communication strategy in response to vaccine hesitancy [1]. This problem might be attributed to vaccinology being traditionally considered a part of theoretical teaching, focusing mostly on lectures and multimedia presentations. Other studies highlight that a practical way of teaching the subject may prove to be much more beneficial [18]. The advantage of such a practical approach to teaching is the fact that it requires both more preparation and interaction; moreover, teaching in smaller groups makes it easier for the students to ask questions and for the teacher to check progress.

Our findings indicate that willingness for a swift vaccination against SARS-CoV-2 among MS depends more on the year of studies than on their grading in vaccinology-related subjects. As many studies regarding the vaccination behaviors and attitudes among MS are restricted to a specific year of study, this link has been not properly investigated [1,2,3]. Nevertheless, previous papers exploring this subject are in concordance with our observations [5]. These findings suggest that students with a more general experience including a closer clinical focus may have a better sense of responsibility for themselves and others.

### 4.2. Personal Experiences during SARS-CoV-2 Pandemic

Due to the nature of their work and their frequent contact with infected patients and infectious materials, doctors and MS are much more prone to infections [19,20]. As such, they are typically tested more frequently, especially using PCR-based tests [21]. Interestingly, despite being more exposed to the virus and receiving more tests, the number of positive tests obtained by the two groups was at a comparable level (MS: 59 (8.59%) vs. NMS: 95 (7.39%), *p* = 0.349).

Despite the fact that, due to their regular contact with the illness, MS might be considered to be more likely to expose their relatives to SARS-CoV-2 infection, this did not appear to be the case: 58.81% of MS relatives suffered from COVID-19 compared to 57.80% of NMS relatives (*p* = 0.736). This lack of discrepancy can perhaps be attributed to the better sanitary regime regarding relatives applied by the MS [22,23]. This in turn can be supported by the fact that MS were more likely to report fearing infecting their elderly relatives than the NMS group, even those who had recovered from COVID-19. This results in a significant reduction in the number of visits: no visits or at most 1 per month, 398 (58.36%) for MS vs. 656 (51.7%) for NMS. This further translates into lower mortality among the relatives, which was found to be around 7% (*p* = 0.889) for both groups.

The fear of infecting or losing a relative to SARS-CoV-2 virus was the greatest fear reported by MS, followed by post-COVID syndrome, general condition deterioration, and professional/academic issues. Both groups of respondents are similarly afraid of the deterioration of their health. The big differences concern the fear of professional/academic problems. This may be related to the fact that students of medical faculties were still participating in classes conducted in hospitals during the study, and the stress caused by learning and exams [24,25,26]. During the COVID-19 pandemic, most of the classes and exams in Poland were conducted online. This change may negatively affect the teaching process, especially the training of MS: the required limitation of practical classes in hospitals disrupts their medical education [27], and could result in the student failing a semester for administrative reasons [28].

### 4.3. The Current Experience and Anxiety Related to Vaccination

The vast majority of the students who took part in the survey reported a willingness to be vaccinated against the SARS- CoV-2 virus. It appears that MS are much more willing to receive the vaccination in comparison to NMS. However, it must be borne in mind that the percentage of those willing to be vaccinated, i.e., higher than 90% in the case of MS, may not be representative of the general population; students who want to receive the vaccination are probably more willing to take part in the questionnaire study. These results are more promising than those obtained in an American survey between 16 and 20 April 2020 testing attitudes among U.S. adults toward a potential SARS-CoV-2 vaccine: only 57.6% were determined to get a vaccine, 10.8% refused vaccination, 31.6% were not sure [29].

The MS appear to have significantly lower fears with respect to vaccination. It has been proposed that this may be due to their greater knowledge and realization in this area acquired in the educational process [17]. Still, regardless of the group, the strongest fears among all the respondents were those concerning the long-term effects of vaccines. Following this, MS reported concerns about the short-term side effects such as fever or malaise. The NMS appeared to be more concerned about what is regarded as conspiracy theories, often propagated by vaccination skeptics: the alleged association between vaccination and autism, restrictions on personal liberties, the chance of microchip implantation, governments’ attempt to control the birth count [30]. Unfortunately, advocates for these theories can also be found among MS; for example, both groups report concerns about the association between vaccination and autism at a comparable level. It may lead to a decline in vaccination rates [31,32]. The spread of such theories is most likely a result of insufficient vaccinology classes throughout the whole education process as well as the wide reach of conspiracy theories on social media [17,33,34].

Injection site reactions and systemic adverse effects, such as headache/lethargy, joint pain, and fever remain the most commonly reported adverse events [35], which is in concordance with data obtained in our survey study (see Table 3). Moreover, MS have a full set of mandatory vaccinations and more often decide to receive recommended vaccinations (e.g., for influenza) [36]. However, the two groups are equally in favor of the complete vaccination package recommended for children according to the vaccination calendar [17].

### 4.4. The Factors Influencing Pro-Vaccination Attitudes

Interestingly, the academic year that the student is attending had a strong influence on their willingness to undergo vaccination: year 3 (2–5) vs. year 2 (1–4; *p* < 0.001). However, the grades which the students received in subjects highlighting the role of vaccination did not have such a strong influence: microbiology (*p* = 0.877); infectious disease course (*p* = 0.743); introduction of pediatrics (*p* = 0.524). This highlights the hypothesis that clinical experience gained by active educational approaches (such as case-based, hands-on practical clinical placements or serious games) are more effective at forming appropriate attitudes and behaviors among MS than the provision of inadequate theoretical knowledge and overrepresentation of lectures [1,2].

In both groups, the willingness for a swift vaccination can be attributed to the following factors: the fear of COVID-19 infection (*p* < 0.001), the level of fear of relatives being infected (*p* < 0.001), the fear of possible vaccine side effects (*p* < 0.001), the general level of fear (*p* = 0.002) and stress (*p* < 0.001) in the last seven days. There appears to be a wide spectrum of public fears in general, both legitimate and unjustified [32]. For this reason, we encourage an objective public debate based on researched and postulated strategies that will help to dispel unjustified concerns [37].

Our findings do not indicate any significant link between anxiety and a willingness to receive a vaccination. This dependency appears to vary between previous studies. Yu et al. noticed that higher anxiety levels during the COVID-19 pandemic did not decrease the vaccination coverage, which prevented a surge in vaccine-preventable disease incidence [38]. Moreover, some studies suggest that a higher anxiety level increased the determination to vaccinate and strengthened the feeling that vaccination was the correct course of action [39]. In contrast, some researchers have noticed that high maternal anxiety levels may result in an increased risk of incomplete vaccination status in infants. For example, Ozkaya et al. assessed this odds ratio as 4.35 (95%CI: 1.87–8.79); statistically significant results were observed even after controlling for sociodemographic factors [40]. These differences may be associated with variations in intensities of fear of infectious diseases and possible side-effects of vaccination. The difficulties may be also strengthened by the existence of a spectrum of childhood diseases which may be triggered and/or exacerbated by infectious agents but also vaccinations, e.g., juvenile idiopathic arthritis [41,42].

The results show that the willingness to vaccinate rapidly was not affected by the severity of depressive symptoms during the previous seven days (*p* = 0.957) and the monthly outgoings (*p* = 0.137). Previous studies examining the relationships between depression symptoms and vaccinations found that even a modest number of depressive symptoms may sensitize the inflammatory response system in older adults and produce amplified and prolonged inflammatory responses after infection, as well as other immunological challenges [43]. Fortunately, depression symptoms did not change significantly after vaccination against influenza [43].

## 5. Conclusions

The medical student group (MS) demonstrated a greater desire to get vaccinated against SARS-CoV-2, and this may be due to their greater health awareness. The willingness to get vaccinated as soon as possible is significantly amplified by fear of passing on the disease to relatives and the year of medical study. In contrast, a fear of vaccination side effects and depression symptoms in the previous week reduce this willingness.

It also appears that COVID-19 vaccine conspiracy theories are less popular among MS. However, both MS and non-medical students (NMS) demonstrate the unfounded concern that vaccines cause autism (~5%).

## 6. Strength and Limitations

This is one of the very first studies, both in Poland and worldwide, exploring the attitudes and behaviors towards SARS-CoV-2 vaccination among students. The data were obtained during the public debate on SARS-CoV-2 vaccination. A sample size calculation was performed. The median depression, anxiety, and stress levels were assessed in one-week on a large group of patients.

We note the following limitations of our study. Firstly, it was an online survey accessed through a link. Thus, only people with Internet access could take part in our study. Moreover, we were not able to calculate the response rate (we do not know how many MS might answer the questionnaire). Secondly, the students who want to receive the vaccination may be over-represented in the questionnaire study; this may affect the results concerning the numbers of MS/NMS who want to get vaccinated. Moreover, as this was only a nationwide study, the obtained results cannot be extrapolated to other groups or nations. Furthermore, the study does not consider the impact of commonly-used drugs on participants’ mental well-being [44].

## Figures and Tables

**Table 1:** Study and control group characterization (*n* = 1971).

	Medical Students	Non-Medical Students
Total; *n*	687	1284
Male; *n* (%)	242 (35.23%)	728 (56.70%)
Median age	21 (20–24)	20 (19–22)
Population of the place of employment/study; *n* (% of complete data):		
City > 500,000 residents	502 (75.83%)	588 (50.56%)
City > 250,000 residents	69 (10.42%)	175 (15.05%)
City > 100,000 residents	29 (4.38%)	143 (12.3%)
City > 50,000 residents	14 (2.12%)	63 (5.42%)
City < 50,000 residents	23 (3.48%)	126 (10.83%)
Countryside	25 (3.77%)	68 (5.84%)
Place of residence as a child; *n* (% of complete data)		
City > 500,000 residents	145 (21.90%)	215 (18.49%)
City > 250,000 residents	50 (7.55%)	87 (7.48%)
City > 100,000 residents	74 (11.18%)	155 (13.33%)
City > 50,000 residents	72 (10.87%)	121 (10.4%)
City < 50,000 residents	151 (22.80%)	296 (25.45%)
Countryside	170 (25.70%)	289 (24.85%)

**Table 2:** Experiences with COVID-19 and related anxiety among medical and non-medical students.

	Medical Students	Non-Medical Students	*p*-Value
Previous SARS-CoV-2 infection	59 (8.59%)	95 (7.39%)	0.349 (Chi2)
Tested for SARS-CoV-2	296 (43.1%)	197 (15.34%)	**<0.001 (chi2)**
Tested for SARS-CoV-2:			
PCR:			
Nose	110 (16.01%)	52 (4.05%)	**<0.001 (chi2)**
Mouth	61 (8.88%)	42 (3.27%)	**<0.001 (chi2)**
Mouth and nose	139 (20.23%)	82 (6.39%)	**<0.001 (chi2)**
Quick antigen test	34 (4.95%)	24 (1.87%)	**<0.001 (chi2)**
ELISA	47 (6.84%)	24 (1.87%)	**<0.001 (chi2)**
Family member with confirmed SARS-CoV-2 infection	404 (58.81%)	745 (57.80%)	0.736 (chi2)
Family member deceased in the course of COVID-19:	47 (6.84%)	90 (7.00%)	0.889 (chi2)
How often do you visit elderly family members?			
Never	95 (13.93%)	213 (16.81%)	0.096 (chi2)
<1×/month	303 (44.43%)	443 (34.96%)	**<0.001 (chi2)**
1–2×month	156 (22.87%)	312 (24.63%)	0.388 (chi2)
3–10×/month	88 (12.9%)	184 (14.52%)	0.325 (chi2)
>10×/month	40 (5.87%)	115 (9.08%)	**0.013 (chi2)**
Fear of contracting SARS-Cov-2 on a 10-point scale:			
General	5 (3–6)	4 (2–6)	**<0.001 (UMW)**
After illness	4 (3–6)	3 (2–5)	0.055 (UMW)
Main COVID-19-related concerns			
Health or academic problems	283 (41.19%)	341 (26.56%)	**<0.001 (chi2)**
Health deterioration	292 (42.50%)	513 (39.95%)	0.272 (chi2)
Post-COVID syndrome	369 (53.71%)	564 (43.93%)	**<0.001 (chi2)**
Health deterioration in family members	510 (74.24%)	809 (63.01%)	**<0.001 (chi2)**
Social stigma	32 (4.66%)	104 (8.10%)	**0.004 (chi2)**
How concerned are you about passing on the disease to your relatives on a scale of 0–10?			
Overall	8 (6–8)	7 (5–8)	**<0.001 (UMW)**
After illness	8 (6–9)	7 (4–8)	**<0.001 (UMW)**

Bold—statistically significant results. Legend: chi2—chi-square test, Fisher—Fisher’s exact test, Yates—Yates-corrected chi-square, UMW—Mann–Whitney U-test.

**Table 3:** Vaccination-related experiences among medical and non-medical students.

	Medical Students	Non-Medical Students	*p*-Value
Do you plan to get vaccinated?			Yes vs. no
Yes–overall	632 (91.99%)	763 (59.42%)	**<0.001 (chi2)**
As soon as possible	524 (76.27%)	389 (30.3%)	As soon as
At some point in the future	90 (13.1%)	374 (29.13%)	possible vs. at
No	28 (4.08%)	279 (21.73%)	some point in the future:
I do not know	27 (3.93%)	242 (18.85%)	**<0.001 (chi2)**
How much are you worried about vaccination side effects on a scale of 0–10?			
overall	2 (1–5)	4 (1–7)	**<0.001 (UMW)**
After previous SARS-CoV-2 infection	2 (1–4)	5 (1–7)	**0.002 (UMW)**
What are you most concerned about regarding vaccination?			
Severe hypersensitivity reaction	66 (9.61%)	88 (6.85%)	**0.030 (chi2)**
Fever and malaise	98 (14.26%)	176 (13.71%)	**<0.001 (chi2)**
Swelling and reddening around point of injection	27 (3.93%)	71 (5.53%)	0.112 (chi2)
Long-term complications	273 (39.74%)	594 (46.26%)	**0.005 (chi2)**
Conspiracy theories (overall):	59 (8.59%)	251 (19.55%)	**<0.001 (chi2)**
Microchip injection	12 (1.75%)	67 (5.22%)	**<0.001(Yates)**
Belief that herd immunity does not exist	6 (0.87%)	35 (2.73%)	**0.061 (Yates)**
Limitation of civil rights	17 (2.47%)	150 (11.68%)	**<0.001 (chi2)**
Control of births by vaccine manufacturers	5 (0.73%)	51 (3.97%)	**<0.001(Yates)**
Autism	27 (3.93%)	70 (5.45%)	0.137 (chi2)
Have you ever experienced any vaccination side effects?	146 (21.25%)	234 (18.22%)	0.105 (chi2)
If so, which one of the following:			
Local reaction	88 (12.81%)	63 (4.91%)	**<0.001 (chi2)**
Fever, malaise	92 (13.39%)	133 (10.36%)	0.436 (chi2)
Severe reaction	14 (2.04%)	39 (3.04%)	0.246 (Yates)
Long-term side effects	10 (1.46%)	46 (3.58%)	**0.007(Yates)**
I do not remember	2 (0.29%)	26 (2.02%)	- (Fisher)
Has anyone from your family experienced any side effects of vaccines?			
Local reaction	88 (12.81%)	63 (4.91%)	**<0.001 (chi2)**
Fever, malaise	92 (13.39%)	133 (10.36%)	**0.044 (chi2)**
Severe reaction	14 (2.04%)	39 (3.04%)	0.326 (Yates)
Long-term side effects	10 (1.46%)	46 (3.58%)	**0.010(Yates)**
I do not remember	2 (0.29%)	26 (2.02%)	- (Fisher)
Past medical history of mandatory vaccinations:			Complete vs rest:
Complete	665 (96.8%)	1155 (89.95%)	
Incomplete	19 (2.77%)	121 (9.42%)	**<0.001 (chi2)**
None	3 (0.44%)	8 (0.62%)	
Past medical history of recommended vaccinations (e.g., influenza one), *n* (%)	248 (36.1%)	354 (27.57%)	**<0.001 (chi2)**
Own children vaccination according to immunization schedule, *n* (%)	30 (88.24%)	51 (77.27%)	0.145 (Fisher)

Bold—statistically significant results. Legend: chi2—chi-square test, Fisher—Fisher’s exact test, Yates—Yates-corrected chi-square, UMW—Mann–Whitney U test.

**Table 4:** (A) Mental well-being according to the DASS-21 questionnaire among medical and non-medical students. (B) Binary logistic regression model assessing the impact of DASS-21 parameters on the willingness to get vaccinated as soon as possible (total: medical and non-medical students).

**A. Mental Well-Being According to the DASS-21 Questionnaire**			
	**Medical Students**	**Non-Medical Students**	***p*-Value**
Depression	6 (3–9)	6 (3–10)	**0.009 (UMW)**
Anxiety	3 (2–6)	3 (1–6)	**0.035 (UMW)**
Stress	7 (4–10)	6 (4–9)	**<0.001 (UMW)**
**B. Binary Logistic Regression Model**			
	**OR**	**95%CI**	***p*-Value**
Intercept	0.713	0.597–0.850	**<0.001**
Depression	0.945	0.920–0.969	**<0.001**
Anxiety	1.006	0.968–1.044	0.757
Stress	1.090	1.054–1.127	**<0.001**

Bold—statistically significant results. Legend: CI—The 95% Confidence Interval, OR—odds ratio, UMW—Mann–Whitney U-test.

**Table 5:** Binary logistic regression model assessing the impact of tested parameters on the willingness to get vaccinated as soon as possible among medical students.

Binary Logistic Regression Model			
	OR	95%CI	*p*-Value
Intercept	1.822	0.467–7.099	0.387
Family member with confirmed SARS-CoV-2 infection (No)	0.995	0.622–1.589	0.984
Family member deceased in the course of COVID-19 (No)	0.847	0.320–2.239	0.737
Past medical history of recommended vaccinations (No)	1.083	0.661–1.773	0.751
The fear of COVID-19 (0–10)	1.110	0.980–1.256	0.101
The fear of passing on the disease to relatives (0–10)	1.255	1.113–1.413	**<0.001**
The fear of vaccination side-effects (0–10)	0.616	0.562–0.675	**<0.001**
Year of medical study	1.270	1.110–1.451	**<0.001**
Depression	0.930	0.867–0.997	**0.043**
Stress	1.068	0.990–1.152	0.086

Bold—statistically significant results. Legend: CI—The 95% Confidence Interval, OR—odds ratio.

## Data Availability

Data are available upon request (Adrian Bartoszek: adrian.bartoszek@stud.umed.lodz.pl).
